# Supplementary material for: Patient and retina specialists’ preferences in neovascular age-related macular degeneration treatment. A discrete choice experiment
Source: PLoS One. 2021 Dec 31;16(12):e0261955. doi: 10.1371/journal.pone.0261955 (PMC8719669; doi:10.1371/journal.pone.0261955)
Supplement: S1 Table — (DOCX) [file pone.0261955.s001.docx]

**Table S1. Patients’ sociodemographic and clinical characteristics**

| Patients’ sociodemographic and clinical characteristics | Patients (N=110) |
| --- | --- |
| Age (years), mean (SD) | 79,0 (7,4) |
| Sex (female), % (n) | 57,3% (63) |
| Smoking habit |  |
| Former smoker (without smoking  >6 months), % (n) | 27,5% (30) |
| Non-smoker, % (n) | 59,6% (65) |
| Smoker, % (n) | 12,8% (14) |
| Not available, n | 1 |
| Presence of concomitant pathology, % (n) | 83,6 (92) |
| Hypertension | 69,1% (76) |
| Peripheral vascular disease | 10,0% (11) |
| Cerebrovascular disease | 3,6% (4) |
| Diabetes | 20,9% (23) |
| Dyslipidaemia | 45,5% (50) |
| Osteoarticular disease | 24,5% (27) |
| Presence of ocular pathology other than AMD, % (n) | **53,6% (59)** |
| Uveitis | 0,9% (1) |
| Previous cataract surgery | 49,1% (54) |
| Glaucoma | 9,1% (10) |
| Diabetic retinopathy | 1,8% (2) |
| Time since AMD diagnosis (years), mean (SD) | 2,3 (0,7) |
| Time since AMD treatment initiation (years), mean (SD) | 2,1 (0,1) |
| Time since anti-VEGF treatment initiation (years), mean (SD) | 2,1 (0.1) |
| OCT: Lesion type |  |
| Type 1 lesion, % (n) | 62,4% (68) |
| Type 2 lesion, % (n) | 23,9% (26) |
| Type 3 lesion, % (n) | 4,6% (5) |
| Mixed lesion, % (n) | 9,2% (10) |
| Not available, n | 1 |
| Presence of atrophy |  |
| Yes, % (n) | 31,8% (35) |
| Not available, n | 1 |
| Presence of fibrosis |  |
| Yes, % (n) | 36,4% (40) |
| Not available, n | 1 |
| anti-VEGF treatment regimen% (n) |  |
| Pro Re Nata (PRN), % (n) | 44,5% (59) |
| Treat and Extend (T&E), % (n) | 44,5% (59) |
| Fixed, % (n) | 4,5% (5) |
| Others, % (n) | 6,4% (7) |
| Number of follow-up visits per patient during study, mean (SD) | 15,6 (5,4) |
| Patients with at least one change of the treatment, % (n) | 48,2% (53) |

AMD: age-related macular degeneration; OCT: optical coherence tomography; retinal angiomatous proliferation; SD: standard deviation;
