# Supplementary material for: Patient and retina specialists’ preferences in neovascular age-related macular degeneration treatment. A discrete choice experiment
Source: PLoS One. 2021 Dec 31;16(12):e0261955. doi: 10.1371/journal.pone.0261955 (PMC8719669; doi:10.1371/journal.pone.0261955)
Supplement: S2 Table — (DOCX) [file pone.0261955.s002.docx]

**S2 Table. List of hospitals / institutions participating in the study**

| Complexo Hospitalario Universitario de Santiago de Compostela |
| --- |
| Hospital Universitario Nuestra Señora de Candelaria |
| Complejo Hospitalario de Navarra |
| Hospital General Universitario de Valencia |
| Hospital Clínico San Carlos |
| Hospital Universitario Araba |
| Hospital Costa del Sol |
| Hospital Universitario Puerto Real |
| Hospital Costa del Sol |
| Hospital Universitario Puerto Real |
| Hospital Punta de Europa |
| Hospital Clínico Universitario de Valladolid |
| Hospital Universitario de Galdakao |
| Hospital Virgen de la Arrixaca |
| Hospital Universitari Son Espases |
| Hospital Universitari Sant Joan de Reus |
| Hospital Universitario Miguel Servet |
| Hospital General de Granollers |
| Hospital Universitari Arnau de Vilanova |
