# Supplementary material for: Patient and retina specialists’ preferences in neovascular age-related macular degeneration treatment. A discrete choice experiment
Source: PLoS One. 2021 Dec 31;16(12):e0261955. doi: 10.1371/journal.pone.0261955 (PMC8719669; doi:10.1371/journal.pone.0261955)
Supplement: Attachment — Submitted filename: Reviwer comments.docx [file pone.0261955.s005.docx]

Dear Editor and Reviewers:

We enclose a revised version of the manuscript "Patient and retina specialists' preferences in neovascular age-related macular degeneration treatment. A Discrete Choice Experiment.".

We appreciate the helpful observations and valuable suggestions of the reviewers. We have addressed the reviewer' comments in the revised manuscript. Modifications are highlighted in track changes. Our responses are listed below (in red) following each specific comment.

**Reviewers' comments:**

Some minor outstanding notes:
Line 58 (track changes copy): What is an "adult naive" patient? Have the authors included the word naive here by mistake?

In this context, an adult naïve is a patient with nAMD whit no previous therapeutic exposure to anti-VEGF treatment.

We have defined naïve patients in the manuscript:

Line 52: “*adult naïve (no previous exposure to anti-VEGF treatment) patients ⩾50 years with confirmed nAMD diagnosis who started anti-VEGF treatment between November 1st, 2016 and February 28th, 2017, with a follow-up of 24 months and not participating in any other clinical study)*”

There is a repeating typographical error (ADM should be AMD) e.g. on lines 82, 97 and elsewhere

Typographical errors have been corrected.

The authors have included their data in the supporting information but it is not in English

Supporting information has been translated into English.
